# Asynchronous recovery of medial and lateral gastrocnemius mechanical properties and jump performance following localized plantar flexor fatigue

**DOI:** 10.1186/s13102-026-01774-x

**Published:** 2026-05-28

**Authors:** Yasemin Şahbaz, Ali İlez, Nergiz Batur

**Affiliations:** 1 Faculty of Health Sciences, Department of Physiotherapy and Rehabilitation, Istanbul Beykent University, Istanbul, Turkey; 2 Department of Human Movement Sciences, Vrije University Amsterdam, Amsterdam, Netherlands; 3 Vocational School, Physiotherapy Program, Istanbul Galata University, Istanbul, Turkey

**Keywords:** Muscle mechanical properties, Gastrocnemius, Fatigue recovery, Countermovement jump, mSEBT, Myotonometry

## Abstract

**Objective:**

To investigate the acute and short-term (24 h) effects of localized plantar flexion fatigue on muscle mechanical properties, countermovement jump (CMJ) kinetics, and dynamic balance, focusing on recovery profiles of the gastrocnemius medialis (MG) and lateralis (LG).

**Design:**

Prospective, repeated-measures experimental study.

**Methods:**

Twenty-eight healthy volunteers (*n* = 28) performed a standardized unilateral isometric plantar flexion fatigue protocol until task failure (Borg CR-10 ≥ 8). Assessments were conducted at baseline, immediately post-fatigue, and 24 h post-fatigue (24 h). Outcomes included myotonometric properties (tone, stiffness, elasticity, relaxation time, and creep) of the MG and LG via MyotonPRO, CMJ kinetics via force plate, and dynamic balance via the Modified Star Excursion Balance Test (mSEBT).

**Results:**

Significant Time × Region interactions were found for stiffness (*P* = 0.038, ηp² = 0.276) and relaxation time (*P* = 0.024, ηp² = 0.171). Both muscle heads showed an acute decrease in stiffness (*P* < 0.001); however, LG stiffness recovered by 24 h (*P* = 0.330), while MG stiffness remained reduced (*P* < 0.05). Although CMJ height recovered at 24 h (*P* = 0.842), eccentric and braking phase durations remained prolonged (*P* < 0.001; ηp² = 0.357 and ηp² = 0.264), indicating altered neuromuscular strategies. For dynamic balance, a delayed impairment was observed in anterior reach distance, which declined only at 24 h compared to baseline (*P* = 0.021, ηp² = 0.133).

**Conclusion:**

Localized plantar flexion fatigue induces region-specific mechanical alterations and asynchronous recovery. Restoration of jump height at 24 h masks persistent deficits in eccentric phase durations and MG stiffness. These findings highlight a “neuromuscular vulnerability window” at 24 h post-fatigue, where delayed impairments in MG mechanics and anterior stability may increase potential susceptibility to injury despite apparent recovery. Practically, monitoring head-specific muscle mechanics, rather than global performance outcomes, is critical during this 24 h window to optimize training modification and guide objective return-to-play decisions.

**Trial registration:**

NCT07457398 (Registration date: February 25, 2026).

## Findings

Localized plantar flexion fatigue induces asynchronous recovery between muscle heads and performance metrics. While vertical jump height returns to baseline within 24 h, medial gastrocnemius stiffness remains significantly reduced and eccentric jump phase durations remain prolonged.

## Implications

Global performance metrics, such as jump height, may mask persistent mechanical and kinetic deficits. Clinicians should monitor phase-specific jump kinetics and head-specific muscle stiffness to more accurately assess neuromuscular recovery and readiness for return to high-intensity activity.

## Caution

These findings were observed in a healthy, recreationally active population following a standardized isometric fatigue protocol. Recovery profiles and the “neuromuscular vulnerability window” (NVW) may differ in elite athletes, clinical populations, or following different fatigue modalities (e.g., eccentric or high-impact loading).

## Introduction

Muscle fatigue is a complex, multifactorial neuromuscular phenomenon characterized by a reversible decline in force-generating capacity, involving a cascade of physiological alterations ranging from ionic imbalances to impaired motor output [1]. The gastrocnemius muscle plays a pivotal role in locomotor performance, not only by generating propulsive power during the terminal phase of gait but also by efficiently storing and releasing elastic energy during the stretch-shortening cycle (SSC) [2]. When localized fatigue develops, the mechanical efficiency of the muscle-tendon unit diminishes, directly limiting explosive power and vertical velocity [3]. Muscle mechanical properties—encompassing parameters such as stiffness, tone, and elasticity—serve as key determinants of force transmission efficiency and energy storage–return capacity. While traditional assessments predominantly focus on active contractile capacity, myotonometry has emerged as a reliable, non-invasive, and objective method to quantify these intrinsic mechanical properties [4]. Recent evidence suggests that myotonometric parameters are highly sensitive to fatigue-induced neuromuscular alterations in athletic populations; however, the temporal relationship between these mechanical changes and functional recovery remains under-explored [4]. Despite the individual importance of muscle mechanics and functional performance, a significant gap remains in the literature. To the best of our knowledge, no previous research has concurrently quantified head-specific (medial vs. lateral) gastrocnemius mechanical properties alongside directional dynamic balance and explosive performance within a repeated-measures fatigue-recovery design. Furthermore, the precise impact of localized fatigue on specific mSEBT reach directions remains highly controversial, with fatigue-induced biomechanical changes often linked to altered neuromuscular control and increased injury susceptibility [5–7]. Clarifying the relationship between these regional mechanical alterations and the time-course of functional recovery in moderately active individuals is essential for evidence-based load management and reducing musculoskeletal vulnerability.

Since gastrocnemius hyper-stiffness and fatigue-related mechanical alterations are known contributors to Achilles tendon pathologies, monitoring these parameters is critical for preventing conditions such as Achilles tendinopathy or calf strains [8–11]. In this context, determining whether tissue mechanical recovery precedes or lags behind functional restoration is crucial to identify the exact NVW, thereby providing safer and more objective return-to-sport decisions.

Physiologically, the MG and LG exhibit distinct architectural and histochemical profiles that justify distinct susceptibility to fatigue and recovery. The MG possesses a significantly larger physiological cross-sectional area, a greater pennation angle, and a higher proportion of fatiguable Type II muscle fibers compared to the LG, rendering the medial head more prone to localized structural micro-trauma and prolonged mechanical degradation following high-intensity tasks. Therefore, this study aimed to investigate the acute and short-term (24 h) effects of localized plantar flexion fatigue on muscle mechanical properties, dynamic balance, and vertical jump performance. Based on these head-specific structural differences, we hypothesized that:

Fatigue would induce region-specific alterations in muscle mechanical properties, with the gastrocnemius medialis (MG) exhibiting more pronounced and prolonged impairment compared to the gastrocnemius lateralis (LG);

Vertical jump performance would show an acute decline but recover within 24 h, potentially masking persistent sub-clinical mechanical deficits;

Dynamic balance, specifically in the anterior direction of the mSEBT, would exhibit a delayed impairment at 24 h, coinciding with persistent deficits in MG stiffness.

## Materials and methods

### Study design and ethical approval

The study protocol was approved by the Istanbul Beykent University Non-Interventional Clinical Research Ethics Committee (Date: 26.11.2025; Meeting No: 2025/10; Document No: E-45778635-050.99-213503). All procedures were conducted in accordance with the principles of the Declaration of Helsinki, and written informed consent was obtained from all participants prior to inclusion. All participants provided written informed consent prior to the commencement of the study. This study was prospectively registered at ClinicalTrials.gov (Unique Protocol ID: UBeykent-17. CTN: NCT07457398).

### Participants

**Fig. 1:**
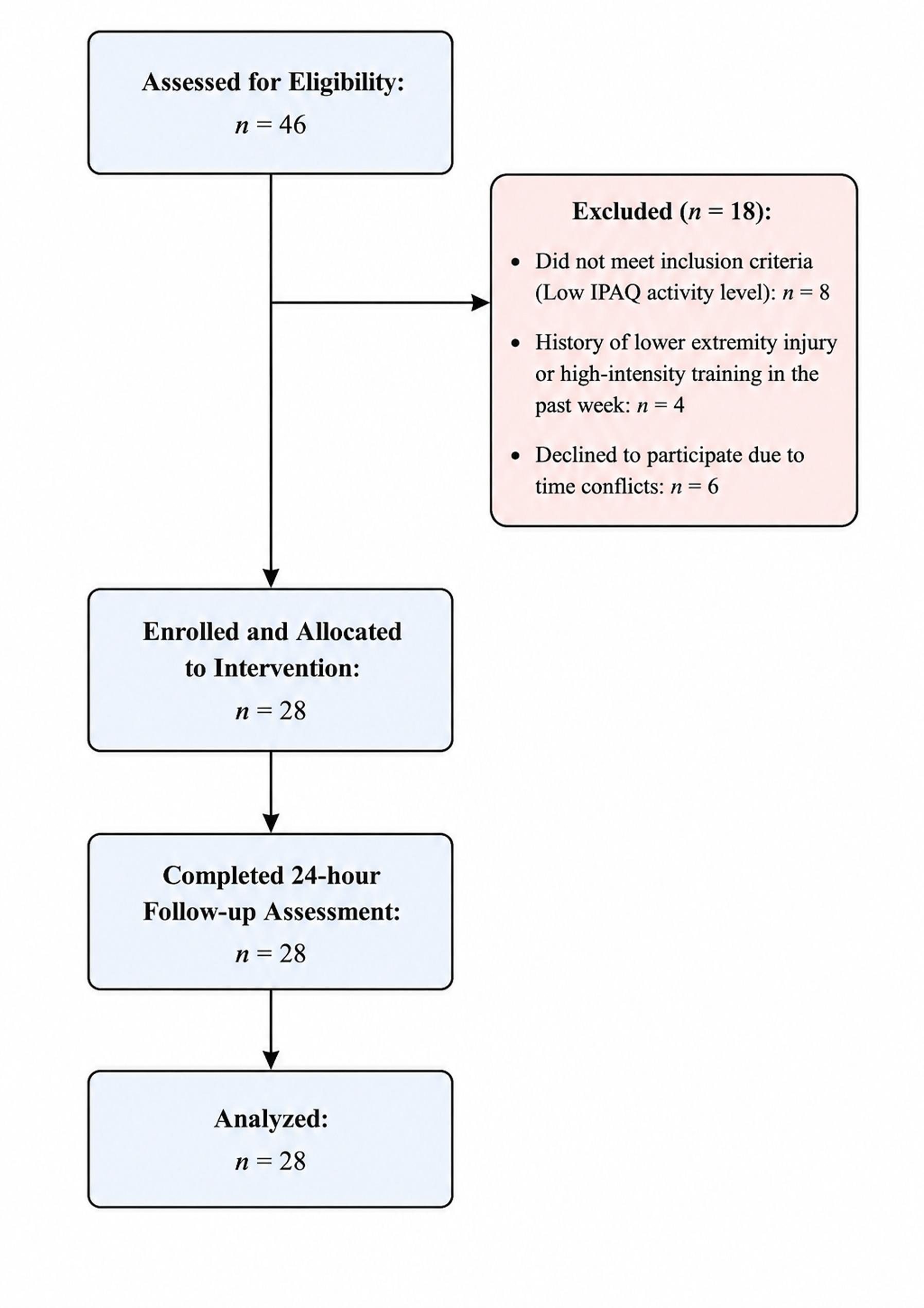
CONSORT flow diagram of the study selection process

A total of 28 healthy volunteers completed the study protocols (Fig. 1). The sample size was estimated using G*Power software (v3.1.9.7). Based on an a priori power analysis for a two-way repeated measures ANOVA (Time [3] × Muscle Region [2]) with a medium effect size (Cohen’s f = 0.25), an alpha of 0.05, and 80% power, the minimum required sample size was determined to be 28. Inclusion criteria were: (a) age 18–30 years; (b) no history of lower extremity musculoskeletal injury in the last six months; (c) a moderate physical activity level (IPAQ category 2).

### Procedures

Assessments were conducted at three time points: Baseline (Pre), immediately post-fatigue (Post-0), and 24 h post-fatigue (Post-24 h). All measurements were performed in a climate-controlled laboratory at the same time of day to minimize circadian influence. To ensure consistency, the same investigator performed all assessments, and participants performed functional tests barefoot. The assessment order was standardized as Muscle Mechanical Properties (Myotonometry), CMJ, and mSEBT to prioritize capturing acute alterations.

### Fatigue protocol

Localized muscular fatigue was induced using a standardized unilateral isometric plantar flexion protocol applied to the dominant limb in a supine position with the foot positioned against a solid vertical surface. Participants were required to perform a sustained continuous maximal voluntary isometric contraction (MVIC) pushing against the surface until task failure. Task failure was defined as a combination of subjective and objective criteria: a score of ≥ 8 on the Borg CR-10 Scale and the objective inability to maintain the required isometric foot position or the required pressure against the surface for more than 3 consecutive seconds, despite standardized verbal encouragement. The investigator closely monitored the participants’ foot alignment and muscle contraction effort to ensure task failure was reached. This protocol has been previously validated to induce localized fatigue and neuromuscular alterations in the gastrocnemius [3, 9]. All post-fatigue measurements were initiated within 30 s of task failure.

### Assessment of muscle mechanical properties

The mechanical properties of the medial (MG) and lateral (LG) gastrocnemius were measured using the MyotonPRO (Myoton Ltd, Estonia). Participants were positioned prone with feet hanging off the edge of the table. Measurements were performed exclusively on the dominant limb. The MG was measured at 30% of the distance between the popliteal crease and the calcaneus, and the LG was measured at the point of maximal muscle belly girth. The recorded parameters included state of tension (Tone/Frequency), dynamic stiffness, elasticity (Logarithmic Decrement), relaxation time, and creep (Deborah number). At each anatomical site, three consecutive measurements were performed, and their average was used for further analysis. To ensure data accuracy, each individual measurement consisted of a train of five consecutive mechanical oscillations, with the mean value of these oscillations being automatically recorded by the device. To eliminate intra-rater variability and measurement error, the device was configured to automatically deliver a series of 5 rapid, mechanical impulses within milliseconds. The internal software of the MyotonPRO automatically averaged these consecutive replicates to provide a single stable value for each measurement session, ensuring maximum data reproducibility without the risk of operational positioning shifts [12].

### Vertical jump performance (CMJ)

Explosive power was evaluated using the Countermovement Jump (CMJ) test. Vertical jump performance was evaluated using the VALD ForceDeesk dual force platform (VALD Performance, Brisbane, Australia). The system collected ground reaction force data at a sampling frequency of 1000 Hz. All kinetic and kinematic data were automatically processed and analyzed using the ForceDeesk software (v2.0). To ensure data quality, the software applied a low-pass Butterworth filter with a 10 Hz cut-off frequency to smooth the raw data. Jump height was calculated using the impulse-momentum method, which is the gold standard for force platform analysis. Participants performed three maximal CMJs with hands on hips and a 60-s rest interval between trials. Kinetic and kinematic parameters analyzed included Jump Height, Relative Peak Power, Peak Force, Take-off Velocity, Flight Time, and Impulse.

### Dynamic balance (mSEBT)

Dynamic balance was assessed using the Modified Star Excursion Balance Test (mSEBT) protocol. To eliminate learning effects, participants performed three practice trials followed by three recorded trials. Reach distances in the anterior (ANT), posteromedial (PM), and posterolateral (PL) directions were recorded. All reach distances were normalized to leg length (%LL) to allow for inter-individual comparison, and a composite score was calculated. To ensure high measurement reliability and minimize inter-tester variability, all assessments were performed by the same experienced investigator, and the standardized mSEBT protocol—which has been previously established as a reliable measure of dynamic balance in this population—was strictly followed. The composite score for the mSEBT was calculated for each limb using the following standardized formula:

$$\text{Composite Score}\:\left(\%\right)=\left(\left(\mathrm{Anterior}+\mathrm{Posteromedial}+\mathrm{Posterolateral}\right)/\left(\mathrm{3}\times\text{Leg Length}\right)\right)\times\mathrm{100}$$

where reach distances and leg length (measured from the anterior superior iliac spine to the medial malleolus) were recorded in centimeters. To minimize measurement error and eliminate potential learning effects, extensive familiarization trials were conducted prior to data collection. The modified Star Excursion Balance Test (mSEBT) is documented to possess high reproducibility and excellent intra-tester reliability in young adults, with established Intraclass Correlation Coefficient (ICC3,1) values ranging from 0.86 to 0.91, ensuring that the delayed deficits captured during recovery reflect true biomechanical phenomena rather than random measurement artifact [5].

### Statistical analysis

Data were analyzed using SPSS (Version 26.0). The dataset was screened for outliers and missing values; no data points were excluded as all participants completed all assessments. Normality was verified via Shapiro-Wilk tests and Q-Q plots. A Two-way Repeated Measures ANOVA (Time [3] × Muscle Region [2]) was utilized for myotonometric data, and a One-way Repeated Measures ANOVA was used for CMJ and mSEBT parameters across the three time points. Post-hoc analyses were performed using Bonferroni correction to adjust for multiple comparisons. Mauchly’s test of sphericity was utilized to verify the sphericity assumption for the repeated measures factors. In instances where the assumption of sphericity was violated (*p* < 0.05), the Greenhouse-Geisser correction was applied to adjust the degrees of freedom and control for Type I error inflation. Effect sizes were reported as partial eta squared (ηp²) for ANOVA (interpreted as: 0.01 = small, 0.06 = medium, 0.14 = large). Significance was set at *p* < 0.05.

## Results

### Participant characteristics

A total of 28 healthy, recreationally active volunteers (18 females, 10 males) successfully completed the experimental protocol. The participants had a mean age of 22.03 ± 1.64 years and a mean BMI of 21.87 ± 2.39 kg/m². According to the IPAQ scores, the physical activity level of the cohort was 1946.57 ± 940.56 MET-min/week, which classifies the participants as recreationally active young adults. Most participants (89.3%) were right-leg dominant. Detailed anthropometric and descriptive characteristics are presented in (Table 1).

**Table 1:** Demographic and physical characteristics of the participants

Continuous Variables	Mean ± SD	Min-Max
Age (years)	22.03 ± 1.64	20.00–25.00
Height (cm)	170.67 ± 6.76	160.00-186.00
Body Mass (kg)	63.78 ± 8.42	46.00–80.00
BMI (kg/m²)	21.87 ± 2.39	16.56–24.91
IPAQ Score (MET-min/week)	1946.57 ± 940.56	396.00-2946.00
Categorical Variables	**n**	**%**
Gender Female Male	18 10	64.3 35.7
Dominant Side Right Left	25 3	89.3 10.7

*SD* Standard deviation, *Min*-*max* Minimum-maximum, *n* Number of participants, *BMI* Body mass index, *IPAQ* International Physical Activity Questionnaire, *MET*-*min*/*week* Metabolic equivalent of task minutes per week

### Muscle mechanical properties of the gastrocnemius

Two-way repeated measures ANOVA revealed significant Time × Region interactions for dynamic stiffness (F = 10.279, *p* = 0.038, ηp²=0.276) and relaxation time (F = 5.556, *p* = 0.024, ηp²=0.171), indicating divergent mechanical responses between the MG and LG following fatigue (Table 2).

**Table 2:** Muscle elastic properties of the medial and lateral gastrocnemius heads across the evaluated time points

Parameter	Region		Pre	Post	24 h	F	pT	pR	pT×*R*	ηp^2^
Tone (Hz)	Med.	Mean ± SD	15.18 ± 2.97	14.46 ± 2.94	14.61 ± 2.46	3.032	0.056	0.708	0.246	0.101
		95% CI	14.03–16.33	13.32–15.60	13.65–15.56					
	Lat.	Mean ± SD	14.88 ± 3.15	14.14 ± 3.27	14.93 ± 2.83					
		95% CI	13.66–16.10	12.87–15.40	13.83–16.02					
Stiffness (N/m)	Med.	Mean ± SD	305.71 ± 92.82	275.57 ± 80.43^a^	278.14 ± 70.02^a^	10.279	**< 0.001**	0.817	**0.038**	0.276
		95% CI	269.7-341.6	244.4-306.6	251.1-305.2					
	Lat.	Mean ± SD	307.78 ± 107.9	269.32 ± 99.27 ^a^	300.35 ± 93.92 ^b^					
		95% CI	266.0-349.5	230.9-307.7	264.0-336.6					
Elasticity (log)	Med.	Mean ± SD	1.21 ± 0.26	1.26 ± 0.22 ^a^	1.24 ± 0.20	10.518	**< 0.001**	0.903	0.443	0.280
		95% CI	1.11–1.31	1.17–1.34	1.16–1.32					
	Lat.	Mean ± SD	1.22 ± 0.33	1.28 ± 0.33 ^a^	1.20 ± 0.22					
		95% CI	1.09–1.35	1.15–1.41	1.11–1.28					
Relaxation (ms)	Med.	Mean ± SD	17.47 ± 4.14	18.48 ± 4.73	18.34 ± 3.19	5.556	**0.007**	0.432	**0.024**	0.171
		95% CI	15.86–19.07	16.64–20.31	17.10-19.57					
	Lat.	Mean ± SD	17.92 ± 4.79	19.30 ± 5.69 ^a^	17.60 ± 3.74					
		95% CI	16.07–19.77	17.10–21.50	16.16–19.05					
Creep (De)	Med.	Mean ± SD	1.10 ± 0.16	1.15 ± 0.15 ^a^	1.13 ± 0.13	4.717	**0.013**	0.795	0.170	0.149
		95% CI	1.03–1.16	1.09–1.20	1.07–1.18					
	Lat.	Mean ± SD	1.11 ± 0.21	1.16 ± 0.21 ^a^	1.09 ± 0.14					
		95% CI	1.03–1.19	1.07–1.24	1.03–1.14					

*Pre* Pre-fatigue, *Post* Post-fatigue;, *24 h* 24 h post-fatigue, *Med*. Medial head of the gastrocnemius, *Lat*. Lateral head of the gastrocnemius, *pT* Main effect of time, *pR* Main effect of region, *pTxR* Time × region interaction, *ηp*^*2*^ Partial eta squared, *Hz* Hertz (frequency), *N/m* Newton per meter, *log* Logarithmic decrement, *ms* Milliseconds, *De* Deborah number. F values represent the test statistics from the Two-Way Repeated Measures ANOVA. Adjustment for multiple comparisons: Bonferroni

Post-hoc comparisons (simple main effects):

a) Significant difference according to Pre value (*p* < 0.05); b: Significant difference according to Post value (*p* < 0.05)

Post-hoc analysis demonstrated that both MG and LG exhibited a significant decrease in stiffness immediately post-fatigue (*p* < 0.001). However, recovery patterns differed between regions: LG stiffness returned to baseline values at 24 h (*p* = 0.330), whereas MG stiffness did not show a statistically significant recovery compared to the immediate post-fatigue value.

Relaxation time significantly increased in the LG immediately post-fatigue (*p* = 0.013) and returned to baseline at 24 h (*p* = 0.039). No significant temporal changes were observed for MG relaxation time (*p* > 0.05).

Significant main effects of time were found for elasticity (F = 10.518, *p* < 0.001, ηp²=0.280) and creep (F = 4.717, *p* = 0.013, ηp²=0.149). Both parameters increased immediately post-fatigue; however, post-hoc comparisons did not reveal consistent pairwise differences across time points.

No significant main effects or interactions were observed for muscle tone (*p* > 0.05).

### Vertical jump performance (CMJ)

One-way repeated measures ANOVA revealed a significant main effect of time for Jump Height (F = 11.411, *p* < 0.001, ηp²=0.297), Eccentric Duration (F = 15.018, *p* < 0.001, ηp²=0.357), and Braking Phase Duration (F = 9.689, *p* < 0.001, ηp²=0.264).

Post-hoc comparisons showed that Jump Height significantly decreased immediately after fatigue compared to baseline (*p* < 0.05) but returned to baseline values by 24 h. In contrast, Eccentric Duration and Braking Phase Duration exhibited a different trajectory; both parameters were significantly prolonged at 24 h post-fatigue compared to baseline (both *p* < 0.05). Notably, Eccentric Duration at 24 h was also significantly higher than the immediate post-fatigue values (*p* < 0.05).

No significant changes were observed in RSI-modified, Concentric Peak Velocity, or Peak Power (*p* > 0.05). These results indicate that while functional output (jump height) recovered within 24 h, the underlying movement strategy remained altered.

### Dynamic balance performance (mSEBT)

A significant main effect of time was observed only for the Anterior reach direction (F = 4.218, *p* = 0.021, ηp²=0.133). Pairwise comparisons revealed a delayed impairment pattern; anterior reach distances did not decline significantly immediately post-fatigue (*p* > 0.05) but were significantly reduced at 24 h compared to both baseline (*p* = 0.025) and immediate post-fatigue levels (*p* = 0.023).

In contrast, no significant main effects of time, limb, or interactions were detected for Posteromedial, Posterolateral, or Composite scores (*p* > 0.05). These results suggest that gastrocnemius fatigue specifically impacts postural stability in the sagittal plane with a delayed onset, with no differences between limbs (Tables 3 and 4).

**Table 3:** Vertical jump performance outcomes and phase-specific kinetics across the evaluated time points

Parameter		Pre	Post	24 h	F	pT	ηp^2^
Jump Height (cm)	Mean ± SD	27.06 ± 5.23	25.96 ± 4.77^a^	26.96 ± 4.50	11.411	**< 0.001**	0.297
	95% CI	25.03–29.09	24.11–27.81	25.21–28.71			
RSI-modified (m/s)	Mean ± SD	0.35 ± 0.08	0.34 ± 0.09	0.34 ± 0.08	0.370	0.692	0.014
	95% CI	0.31–0.38	0.31–0.37	0.31–0.37			
Concentric Peak Velocity (m/s)	Mean ± SD	2.21 ± 0.46	2.07 ± 0.40	2.09 ± 0.29	3.127	0.052	0.104
	95% CI	2.03–2.39	1.91–2.23	1.98–2.20			
Eccentric Duration (ms)	Mean ± SD	301.64 ± 75.7	322.14 ± 88.1	371.53 ± 91.4 ^a, b^	15.018	**< 0.001**	0.357
	95% CI	272.2-331.1	287.9-356.4	335.9-407.1			
Braking Phase Dur. (ms)	Mean ± SD	251.0 ± 103.3	271.7 ± 101.9	300.4 ± 100.0 ^a^	9.689	**< 0.001**	0.264
	95% CI	210.8-291.1	232.1-311.3	261.5-339.2			
Peak Power (W)	Mean ± SD	2180.2 ± 628.3	2048.8 ± 621.6	2095.5 ± 527.1	1.137	0.336	0.080
	95% CI	1936.1-2424.3	1807.8-2289.8	1891.0-2300.1			

*Pre* Pre-fatigue, *Post* Immediately post-fatigue, *24 h* 24 h post-fatigue, *F* ANOVA F-value, *pT* Main effect of time, *ηp*^*2*^ Partial eta squared, *RSI*-*mod* Modified reactive strength index, *CI* Confidence interval

Post-hoc comparisons (simple main effects):

Data are presented as mean ± SD. pT: p-value for the main effect of time. ηp²: Partial eta squared

a Denotes a significant difference compared to Pre (*p* < 0.05)

b Denotes a significant difference compared to Post (*p* < 0.05)

**Table 4:** Dynamic postural stability scores during the modified star excursion balance test across time points and limbs

Direction	Limb		Pre	Post	24 h	F	pT	pL	pT×L	ηp^2^
Anterior (cm)	Dom.	Mean ± SD	88.22 ± 10.4	87.66 ± 10.1	86.48 ± 9.5^a, b^	4.218	**0.021**	0.672	0.301	0.133
		95% CI	84.1–92.2	83.7–91.6	82.7–90.1					
	Non-Dom.	Mean ± SD	87.39 ± 9.5	89.15 ± 9.4	84.75 ± 8.0 ^a, b^					
		95% CI	83.6–91.1	85.4–92.8	81.6–87.8					
Posteromedial (cm)	Dom.	Mean ± SD	117.96 ± 11.2	119.57 ± 11.1	119.31 ± 9.9	0.195	0.823	0.781	0.627	0.007
		95% CI	113.5-122.3	115.2-123.9	115.4-123.1					
	Non-Dom.	Mean ± SD	118.89 ± 12.8	118.43 ± 12.6	118.24 ± 8.6					
		95% CI	113.9-123.8	113.5-123.3	114.9-121.5					
Posterolateral (cm)	Dom.	Mean ± SD	107.24 ± 15.8	108.07 ± 13.6	109.42 ± 13.9	0.308	0.736	0.187	0.508	0.011
		95% CI	101.1-113.3	102.7-113.3	104.0-114.8					
	Non-Dom.	Mean ± SD	109.96 ± 14.8	108.60 ± 13.5	109.86 ± 16.1					
		95% CI	104.1-115.6	103.3-113.8	103.6-116.1					
Composite Score (%)	Dom.	Mean ± SD	104.4 ± 10.6	105.1 ± 9.1	105.0 ± 9.7	0.545	0.583	0.887	0.781	0.020
		95% CI	100.3-108.6	101.5-108.6	101.2-108.7					
	Non-Dom.	Mean ± SD	105.4 ± 10.5	105.4 ± 8.8	104.3 ± 9.0					
		95% CI	101.3-109.5	101.9-108.8	100.8-107.8					

Values are presented as Mean ± Standard Deviation and [95% Confidence Interval]

*Pre* Pre-fatigue, *Post* Immediately post-fatigue, *24 h* 24 h post-fatigue, *Dom*. Dominant limb, *Non*-*Dom*. Non-dominant limb, *pT* Main effect of time, *pL* Main effect of limb, *pT×L* Time × limb interaction effect, *ηp²* Partial eta squared

Composite Score: the average total performance value across the three reach directions

Post-hoc analysis for the Anterior reach direction: Performance values significantly decreased at 24 h compared to both pre-fatigue (*p* = 0.025) and immediately post-fatigue (*p* = 0.023) measurements

No significant differences were observed for Posteromedial, Posterolateral, or Composite Score (*p* > 0.05)

## Discussion

This study investigated the acute and short-term (24 h) effects of localized plantar flexion fatigue on muscle elastic properties, vertical jump performance, and dynamic balance. Our primary findings revealed a regional heterogeneity in the recovery of intrinsic mechanical parameters; the medial gastrocnemius (MG) exhibited persistent stiffness deficits at 24 h, whereas the lateral head (LG) normalized. Furthermore, a significant discrepancy was observed in jump kinetics: while jump height recovered within 24 h, eccentric and braking phase durations remained significantly prolonged. Most notably, dynamic balance impairment in the anterior direction of the mSEBT followed a delayed pattern, manifesting only at the 24 h mark. These results suggest that the recovery of muscle mechanics and neuromuscular control strategies follows an asynchronous and region-specific timeline.

### Regional heterogeneity in mechanical property recovery: MG vs. LG

A key finding was the divergent recovery trajectory of muscle stiffness between the two heads of the gastrocnemius. While both MG and LG showed an immediate post-fatigue decline in stiffness, only the LG normalized at 24 h. This persistent reduction in MG stiffness suggests that the medial head is more susceptible to prolonged fatigue-induced mechanical alterations. This regional heterogeneity is likely rooted in the anatomical and architectural dominance of the medial head. The MG possesses a larger physiological cross-sectional area and a more pennate fiber arrangement, enabling it to carry a greater proportion of the mechanical load during high-intensity plantar flexion tasks compared to the LG [13].

Furthermore, the MG exhibits different motor unit recruitment patterns and a higher metabolic demand during isometric contractions until task failure. The higher mechanical stress placed on the medial head in our protocol might have contributed to structural stress, such as possible myofibrillar micro-trauma or transient alterations in titin filament tension, thereby potentially delaying the restoration of its passive mechanical properties. Since direct histological data or molecular biomarkers were not evaluated, these underlying physiological explanations remain strictly hypothetical possible mechanisms, and our findings should be interpreted primarily through the observed mechanical changes in muscle stiffness and elasticity. These results align with recent evidence suggesting that synergistic muscles within the same group do not fatigue or recover uniformly, necessitating head-specific monitoring in clinical and athletic settings [14].

Furthermore, the utilize of a unilateral isometric protocol inherently influences the load distribution and synergistic sharing between the MG and LG. Even during isolated unilateral tasks, neural drive and mechanical stress are not uniformly distributed across the plantar flexors. The MG, with its larger physiological cross-sectional area and primary contribution to plantar flexion torque, typically handles a disproportionate amount of the initial mechanical load. As the unilateral contraction progresses toward task failure, complex inter-muscular load shifting and compensatory adjustments occur between the two heads, which heavily contextualizes the asynchronous mechanical changes and prolonged tension profiles observed in the MG compared to the LG [9].

### Compensatory neuromuscular strategies masking persistent deficits

Regarding explosive performance, our results demonstrated that while CMJ height recovered within 24 h, evaluating this metric in isolation masks underlying neuromuscular deficits. We observed that eccentric and braking phase durations remained significantly prolonged at 24 h. This phenomenon suggests a compensatory strategy where participants altered their jump mechanics to maintain performance despite compromised muscle-tendon stiffness.

Physiologically, the persistent reduction in MG stiffness likely diminished the efficiency of the stretch-shortening cycle (SSC). To generate the necessary impulse for a baseline-level jump height, participants likely increased the duration of the eccentric and braking phases, allowing for a longer period of force application [15]. This shift in strategy indicates that the “readiness” of the neuromuscular system is not fully restored at 24 h, even if traditional outcome measures like jump height suggest otherwise. This finding is critical for sports practitioners, as it highlights a NVW where altered movement patterns could increase potential susceptibility to musculoskeletal injury. This discrepancy introduces a dangerous “false sense of confidence” for both athletes and clinicians; relying solely on gross functional tests may lead to premature return-to-sport decisions. From an injury mechanics perspective, this “asynchronous recovery”—where global performance normalizes while local mechanical properties lag behind—compromises the muscle-tendon unit’s capacity for optimal shock absorption and energy storage. Consequently, during high-velocity movements, the mechanical stabilization burden shifts onto synergistic structures, potentially increasing the vulnerability to secondary strain or Achilles tendon pathology. Our identification of a NVW at 24 h has direct clinical implications for sports practitioners and physiotherapists. These results suggest that relying solely on the absence of acute fatigue may be misleading for return-to-play timing. Specifically, even when explosive power (CMJ) seems to have fully recovered, the persisting impairments in MG mechanical properties and anterior stability could increase potential susceptibility to injury. Therefore, post-fatigue recovery protocols should extend beyond the immediate recovery phase, incorporating objective assessments of both muscle mechanics and dynamic balance before an individual is cleared for full athletic participation.

### Delayed dynamic balance impairment and mechanofunctional linkage

The delayed impairment in mSEBT anterior reach performance at 24 h contradicts the traditional assumption that balance is most compromised immediately post-fatigue. The anterior reach is uniquely challenging as it requires maximal ankle dorsiflexion while maintaining postural stability through the eccentric control of the plantar flexors [5]. Therefore, the observed deficit at 24 h post-fatigue may be attributed to the specific biomechanical demands of this reach; this mechanofunctional linkage appears to remain compromised during the delayed recovery phase, even after acute fatigue has subsided.

A crucial biomechanical explanation for this delayed deficit lies in the persistent stiffness reduction of the MG. During the anterior reach, the MG-tendon unit must provide critical passive tension support to stabilize the tibia’s forward translation. The significant loss of intrinsic stiffness observed at 24 h could have hypothetically compromised this “passive braking” mechanism, potentially requiring the neuromuscular system to rely more heavily on active eccentric control, which was already suboptimal due to the prior fatigue. This reduction in passive tension support effectively limits the safe range of forward leaning, manifested as a decreased reach distance.

Additionally, this is a possible mechanism that might be linked to altered co-contraction strategies between the gastrocnemius heads and the tibialis anterior (TA). If the mechanical properties are still compromised at 24 h, compensatory co-activation patterns could potentially become inefficient. However, because electromyographic data were not recorded in this study, this interaction represents an unverified, albeit plausible, pathway. Furthermore, the 24 h mark coincides with the typical onset of delayed-onset muscle soreness (DOMS) and potential remodeling-phase depressions in motor unit firing rates, further exacerbating the functional deficit [16].

It is important to note that while the fatigue protocol was applied unilaterally to the dominant limb to ensure localized mechanical control, the observed impairments in dynamic balance were evident bilaterally. This phenomenon suggests that unilateral localized fatigue may induce a crossover effect, likely mediated by central fatigue mechanisms or a global alteration in postural control strategies. Although the mechanical property changes were specific to the fatigued limb, the resulting functional deficits in tasks requiring bilateral coordination, such as the mSEBT, indicate that the neuromuscular system’s ability to manage stability is compromised beyond the localized site of fatigue.

### Limitations and future directions

This study has several limitations that should be acknowledged.

First, the sample consisted of healthy, recreationally active young adults; therefore, these findings may not be directly generalizable to elite athletes who may possess faster recovery kinetics and different compensatory neuromuscular strategies [3, 16].

Second, a distinct methodological limitation is the reliance on the subjective Borg CR-10 scale (score ≥ 8) as the primary task termination criterion, rather than utilizing an objective strength loss threshold, such as a verified 50% maximal voluntary contraction (MVC) reduction. While the Borg scale is a widely accepted and validated clinical tool for capturing perceived exertion and task failure, the lack of simultaneous objective torque monitoring means that the exact physiological quantification of force depreciation at the exact moment of task termination could not be standardized.

Third, while the assessment of muscle mechanical properties via myotonometry provided objective data, our 24-hour follow-up window was insufficient to identify the exact time point of full restoration for the MG.

Fourth, although we focused on the gastrocnemius heads, the potential compensatory role of the soleus or the influence of arm movement during dynamic balance—as highlighted in recent research [7, 3] was not explicitly measured. Future research should examine elite athletic populations, utilize longer recovery windows, combine perceptual metrics with continuous objective force criteria, and integrate high-density EMG or ultrasound elastography to further elucidate the complex relationship between region-specific recovery and functional motor output.

Fifth, due to the mixed-sex sample and the fact that the sample size was not statistically powered to perform a sub-group analysis by sex, sex was not considered as a factor in the main statistical analysis. Future studies with larger, sex-specific cohorts are required to clarify potential sex-based differences in localized muscle fatigue and recovery kinetics.

### Perspective

The present findings challenge the reliance on global performance outcomes, such as jump height, for assessing recovery and “readiness-to-train.” Our data demonstrates a NVW at 24 h post-fatigue, where functional output appears restored but movement strategies (prolonged eccentric and braking phases) and intrinsic tissue properties (medial gastrocnemius stiffness) remain suboptimal. This asynchronous recovery suggests that clinicians and coaches should prioritize phase-specific jump kinetics and head-specific mechanical monitoring over traditional outcome measures. Failure to recognize these masked deficits could lead to premature return-to-play decisions and an increased risk of overuse injuries. These results extend previous biomechanical research by highlighting the regional heterogeneity of the gastrocnemius heads and the delayed onset of dynamic postural instability following localized fatigue, as previously discussed in related neuromuscular studies [9, 14, 17].

## Conclusion

Localized plantar flexion fatigue induces region-specific alterations in muscle mechanical properties and functional performance. A critical finding of this study is the regional heterogeneity in recovery: medial gastrocnemius (MG) stiffness remains significantly compromised at 24 h, whereas the lateral head (LG) normalizes. Furthermore, the apparent recovery of vertical jump height at 24 h masks persistent shifts in neuromuscular strategy, characterized by prolonged eccentric and braking durations.

The delayed impairment in mSEBT anterior reach further underscores a NVW that manifests well after acute fatigue has subsided. However, because this study was conducted on healthy, recreationally active young adults, these findings should be generalized to elite athletes or elderly populations with caution, as recovery kinetics and compensatory strategies may differ significantly. Clinicians, physical therapists, and coaches should not rely solely on global performance metrics like jump height; instead, they should monitor head-specific mechanical parameters and jump phase durations to accurately guide load management and mitigate the risk of lower-limb injuries during the post-fatigue recovery period. Future protocols must incorporate objective, muscle-specific myotonometric monitoring alongside functional metrics to ensure true physiological readiness.

## Data Availability

Due to ethical restrictions regarding participant privacy, the data are not publicly available but can be provided by the corresponding author.

## Abbreviations

GN: Gastrocnemius
MG: Medial Gastrocnemius
NVW: Neuromuscular Vulnerability Window
LG: Lateral Gastrocnemius
mSEBT: Modified Star Excursion Balance Test
CMJ: Countermovement Jump
RPE: Rating of Perceived Exertion
